# Upon the Pathology of Milksickness

**Published:** 1862-05

**Authors:** Geo. Wheeler Jones

**Affiliations:** For the Degree of Doctor in Medicine. Medical Department of Lind University


### CHICAGO MEDICAL EXAMINER.

N. S. DAVIS, M.D., Editor.

VOL. III. MAY, 1862. NO. 5.

### Original Contributions.

ARTICLE XIII.

### UPON THE PATHOLOGY OF MILKSICKNESS.

A Thesis, by Geo. Wheeler Jones,

FOR THE DEGREE OF DOCTOR IN MEDICINE. MEDICAL DEPARTMENT OF LIND UNIVERSITY. SESSION 1861-2.

Gentlemen:—Had it fallen to my lot to write this article but a comparatively short time since, I should certainly have apologized for so doing, as the disease of which I treat was then unclassified, and by some pronounced unknown. It has always been considered a wonderful mystery by the laity, and the opprobria medicinæ of the Western physician; and will continue to be so, until the profession advances to the examination with unbiased minds, and spirits willing to examine the case in all its bearings, and give to *truth* and *facts* their full value. Such has been my effort; and having for several years resided in a "Milksick region"; having had the subject often brought before my mind, I have endeavored to divest it of its shadows, its ogres, and its phantasms—to draw it into the clear light of science, and there analyze it carefully, with reason as my assistant and guide. And here, standing in the outer court of the Æsculapium, with an eager desire to penetrate its "holy of holies," I tender, as my sesame, the result of my thoughts and observations upon this strange disorder, hoping the doors may unfold, and I be received a worthy follower of Chiron's ghost.

The early history of "Milksickness" is involved in much obscurity. I have been unable to find any account of the disease previous to the writings of Dr. DRAKE, and his contemporaries. It—the disease—is found in a "belt of country comprising Ohio, Indiana, and Illinois," and extending into Kentucky and Tennessee. There are also spots in Alabama where it is said to exist. I believe it is unknown West of the Mississippi, North of the great Lakes, and East of the Alleghany range. To Dr. DRAKE is due the honor of having first brought this subject fully before the profession; and although many years have passed since then, its pathology is still, to the mass of the profession, involved in deepest darkness and doubt. Why this is so, may readily be seen, if one will take the trouble to peruse the mass of literature perpetrated on its account. Views as different as light from darkness; weighty conclusions from equally weighty arguments; facts and truths distorted and disfigured until their farther recognition is impossible; strange ideas and wonderful discoveries, all have been inflicted upon a patient, loving, and attentive profession, "until forbearance ceases to be a virtue," and here to the brimmer I will add one more drop.

During the early writings of DRAKE, those who recognized the complaint were divided into two principle factions as to its primary cause; DRAKE and his followers maintaining it was vegetable; his opponents, that it was mineral. The former strongly suspecting the rhus toxicodendron; the latter, arsenic, cobalt, and sulphur. Since then, these two have been divided into four theories, viz.: vegetable, mineral, animal, and malarial. The advocates of the vegetable cause have each found a plant, which he is certain is *the* one. Amongst those suspected are the eupatorium ageratoides, (white snake root,) bignona capreolata, (catalpa,) rhus tox. (poison oak,) rh. radicans, (poison vine,) apocynum cannabinum, (Indian hemp,) and a vegetable fungus, christened by Prof. SLACK, who claims its discovery, ergodeleteria; the last of which was recently largely experimented upon by Dr. NAGLE, a Southerner, who contributed a valuable paper upon the disease in question to the *Nashville Journal*, wherein he ably advocated the claims of the fungi, and supported his views by arguments and conclusions drawn from actual experiment,—which is more than others can maintain. There has also an article recently appeared, in which the claims of cicuta virosa (water hemlock,) are advocated. But there is *no* vegetable, amongst the many named, which has been able to bear the scrutiny of extended examination; not one but that is found growing largely in regions entirely freed from the curse of our disease,—very few but of which cattle may and do eat with impunity. And with such strong negative proof against this theory, I do not think we are justified in upholding it, as it really prevents a calm and careful examination of other views. The same too may be said of the mineral cause; no one of this class of substances, with which we are acquainted, affects the system as does the subtile poison of Milksickness. In the mining regions of Bohemia and Saxony, where cobalt and arsenic occur in great abundance, the disease is unknown; in the sulphurous atmosphere of Solfatara, Sicily, and the Roman States, no Milksickness is found; in the iron producing countries of the globe, no such complaint is known; in short, no mineral can be found which produces a like disorder. The animal theory is most unworthy of them all, for it does not go even to the surface of the subject. If the complaint is caused by eating animal food, what gave it to the animal? It is not a secondary cause for which we seek, that we know well enough; it is the great primary cause which now occupies the mind.

"Can it be of malarial or miasmatic origin?" I not only think it can, but that it is; and will try to advance a few reasons for my belief. I conceive the cause of this disease to be a peculiar miasm, generated under peculiar circumstances. It may be an animal sporule; a vegetable atmospheric fungus; or a gas formed by a particular combination of the elements; that it is one of these three I am confident; but which one, is at present beyond the power of man to determine; although our evidence leans most powerfully toward the first two. We generally, I may say invariably, find Milksickness in or near a notoriously miasmatic district. Northeast of the place in which I reside, the country is low, wet, and might almost be called marshy. North and Southeast it is high and rolling, but very much cut up by transient water courses. The land in all these places is covered with a thick growth of forest trees and underbrush; and in all these spots we have much malarious fevers; and the latter, I am confident, will be found to be the case in all Milksick regions.

During the last fall, 1861, this complaint has been quite marked in that and adjoining counties. The weather was such as we usually have at that season, and such as will generally give rise to miasmatic disorders, with which we had the Milksickness complicated; its most common accompaniment this season was remittent fever. I do not wish it understood here that I advocate the theory that Milksickness is a grade of intermittent or congestive fever; but this I do maintain, that the causes which generate common miasm, will, under certain peculiar circumstances, be totally and thoroughly changed in their combination, producing another substance which will in turn give rise to a totally distinct disease from the one previously rife. How often do we see this in other complaints? Variola and scarlatina will rage in the same district. Yellow fever and cholera will stalk throughout the land side by side,—and as each finds a being peculiarly predisposed to its own special action, on him will fall the blow. Still no one will say that these are each produced by the same cause, nor that they are different grades of the same disease. Each may be caused by an atmospheric fungus, an animal sporule, or a gaseous vapor; but these causes are generated under different conditions of nature's laws, and each produces its own distinct legitimate effects, and these effects are only seen where the conditions of the organic changes in air and earth are such as to favor the propagation of their cause; and where the changes are such as occur in many districts of the West and South, there we see produced the peculiar disease known as Milksickness; while in the neighborhood, nay in the same family, we see the usual fevers of malaria. I give the preference to the animal or vegetable nature of this cause, from the fact that the disease is a self-propagating one, and to some degree contagious, which strongly indicates a cause which will and does generate itself from itself; and we can hardly imagine such a case, without calling to our assistance those laws of nature which are intimately associated with the mysteries of organic life, and there the fact, that it is and must be a microscopic animal, a microscopic fungus, or an organic cell germ, breaks forcibly through the mind, and sheds upon the gloom-shrouded spirit a beam of hope-inspiring light, guiding our steps nearer and nearer to the great truth, which, God grant, we may all soon reach.

I have said this disease is contageous; for the proof of which I give the following facts, viz.: In perhaps seven-eights of the cases which occur in man, the disease can be clearly traced to the eating of the flesh, milk, or butter of an animal which has died of or is sick with the complaint; and in all such cases the same disease is produced, which plainly shows its power of self-propagation; were such not the case, the disease which would *generally* be produced, were any caused at all, by consuming the diseased and unwholesome food, would be a grade of typhoid, typhus, or scrofula; but where it is *invariably* the same disorder with which the animal furnishing the food is afflicted, can we come to any other conclusion than that the disease is of a contageous nature? And what is true of its effects upon man, are the same in regard to the carnivora; as dogs, cats, buzzards, &c.

Again, human beings have been known to contract the complaint in its worst form, after having been engaged in flaying the diseased and dead but yet warm animal, and after having abstained entirely from the use of food by which it could be contracted. The same might be said of animals; but as the proof that they have not eaten the meat can not be conclusive, it would not be a fair example. With such a fact before us, and the vouchers are satisfactory, can we deny that the cause consists of a peculiar germ, which taken into the system, either through the lungs or stomach, there propagates itself until the whole being is contaminated; and without prompt assistance, the poor sufferer quickly glides down the hill of life, and sinks into the dark abyss of eternity. That the cause is of short life is also true; since before it can rise to the height at which man usually breathes, or ere it can travel to a sufficient distance from its origin to reach the system of man, its pernicious influence is destroyed, and its germs dying before they can find a fit place for propagation, are thus rendered inert. That man has taken the disease from the atmosphere is a fact too strongly verified to be disputed, still such cases are not common; but the human race generally receive the disease from a secondary source—the bodies of the diseased animals; which animals, from their mode of life and manner of procuring sustenance, bring their organs of respiration in immediate contact with the live germs of the disease, and with every inspiration of life incorporate with their systems the seeds of death, which, finding a congenial home in their new abode, rapidly accumulate, and weave around their victim the web of disease and death.

I hope, Gentlemen, I have clearly expressed my views as to the cause of this complaint; and will now turn to its nature and anatomical characteristics. What we know of the latter amounts to very little, and gives but a scanty assistance in our search for its first cause. Several post mortem examinations have been made; but little of importance or interest has been discovered. The chief changes were in the stomach, which has generally been found somewhat injected and contracted, especially at the pyloric extremity; which in many instances was permanently and almost totally closed. The lower portion of the intestines have also, generally, an abnormal redness; which may be accounted for by the irritation of the hardened fœces. The injection of the stomach and upper bowels is undoubtedly caused by the violent retching which accompanies the complaint. The contents of the bowels are in a hardened condition, almost entirely devoid of moisture. There is also "an almost complete absence of gas." In animals, the partly digested food in the stomach is formed in "round hard balls" of various sizes, "and the fœces in the large bowels are so dry as that they readily crumble into powder upon the application of slight force." There are also the usual appearances of lesions, and inflammation in the various organs, when these have occurred during the course of the disease. The great difficulties in the way of procuring post mortems, in the sections of country where the disease occurs, the insurmountable prejudice which the people show against such a proceeding, has rendered it difficult and almost impossible to advance our knowledge in this respect. Still, what has been done goes a great way, and is ample evidence of the non-local nature of the complaint. That it is a disease sui generis, I cannot conceive how one acquainted with the disorder, can deny. Its symptoms, its course, and its characteristics all proclaim it a disease in its self. By a great majority of those who are acquainted with its symptoms and course, from practical observation, it is regarded as a disease of the blood; for, as Dr. BYFORD aptly remarks, "Is there any disease but a blood disease that emits a peculiar odor?" And this odor, of which I shall speak again, is one of the principal characteristics of the complaint—having been once perceived is never confounded with any other—many practitioners indeed relying upon it as their great diagnostic sign. I can easily conceive how the blood may become poisoned, degraded, and perverted in its properties—how in this condition, by its pernicious influence upon the great centre of life, it spreads a destroying influence upon all the functions of body, the nervous, the secretory, and muscular in chief. And our knowledge of its course supports this view; but the elementary properties are much perverted, if not decidedly diminished; the brain is disordered in almost all grades of action, from simple irregularities to profound coma; the secretory function in all parts of the system is arrested; the spinal cord is affected as betokened by the persevering backache; the muscles are weakened, and the whole animal economy degraded in its various offices; every thing tends to prove the action of a powerful sedative influence upon the system, which influence springs from the demoralized and degraded condition of the blood,—the great source, in health, of life and activity in all the organs. Some may ask, at this point, why then is the action of the stomach so greatly increased? I would ask in return, is the exhibition which this organ displays of its powers a natural action? Most assuredly not. Its natural action is the secretion of the gastic juice, a peculiar mucus, and that function known as absorption. The gastric follicles are dormant in this affection, while the mucous secretion is more abundant, evidently for the purpose of protecting the organ from the effects of the acrid matter, which most probably finds its way there by exmotic action, although it may indeed be produced by a perverted action of the gastric glands; so may the other matter, which I call mucus; but it is generally considered a true mucus formed by the mucous follicles. Can we come to any other conclusion, with this knowledge, than that the blood is deeply poisoned, and in its continued action is carrying destruction to all the body? While nature, becoming aroused to her danger, puts forth her powers to eliminate the cause of death; but the usual portals of excretion are closed, and her unaided efforts are powerless to force the passage; and now, as danger comes nearer, she turns as a last resort to the stomach, perverts its action, drives through its walls the seeds of destruction, which, by their unwonted presence, cause it to call for muscular power to expel the intruding substance,—the weakened nerves, by a mighty effort, respond to the call, arousing the proper muscles for the labor on hand, and boldly undertake its performance; but, in the great majority of cases, too late to effect its end; and unless assistance from the external world is obtained, nature sinks in despair, and death conquers his victim. But I must hasten in my remarks, as time and space are both limited.

In describing the symptoms and treatment, I shall adopt the plan of dividing the disease into three separate grades or varieties, viz.: simple, inflammatory, and congestive. The symptoms of the first being as follows: a strange feeling of languor, and decided aversion to all exercise, both of mind and body, mark its first onset; this is speedily followed by a want of tonicity throughout the entire system, but is specially exhibited in the stomach and voluntary muscles; there is a want of appetite, but at the same time some nausea if food is withheld beyond the usual time; torpidity of the bowels; palpitation of the heart; depressed pulse, but not much irregularity in beat; cool, pale, and shrivelled skin; stiffness of joints; trembling of limbs; shortness of breath; craving for ardent spirits; vomiting after exertion; oppression of epigastrium; general restlessness, and at night absence of healthy slumber; "sometimes, however, the patient is dull, stupid, and indifferent to surrounding circumstances." These symptoms mark the first appearance of the disease, and may continue for days, or weeks, or months, before the complaint manifests itself in a decided manner; and where proper hygienic rules are observed, and attention paid to the general health, it often stops at this point, and the patient is restored to health without the disease advancing to its more serious grades. But if the above measures are neglected it will sooner or later take upon itself a more active form; and this change is usually preceded by some exciting cause, as fasting, long fatigue, obstinate constipation, or over-exertion. The complaint then presents the following characters: the patient is "seized suddenly with nausea, faintness, and prostration;" violent emesis quickly supervenes, accompanied with urgent thirst and longing for alcoholic drinks and iced water; the vomiting generally occurs at regular intervals, and ends with the ejection of the entire contents of the stomach, which is at first composed of whatsoever the patient may have swallowed during the interval; but as the case advances the character of the ejected matter changes, and is now composed of a "glairy tenacious mucus, very acrid and sour;" and then this mucus mixed with a dark "coffee ground matter," or this dark fluid alone; when this mixture is allowed to stand it separates into a colored solid substance, and a "collection of coagula;" the colored matter effervescing with bicarb. sodæ. In the latter stages of violent cases it closely resembles the matter of black vomit. A dirty white coat covers the tongue, which is loose and flabby; the surface of the body is cool, and is now quite full, and of a leaden color, the capillary and cutaneous vessels being in a state of engorgement; the patient is dull and stupid during the intervals of vomiting; the respiration is slow, embarrassed, and accompanied by a peculiar sighing; the pulse is full, slow, and soft; the heart throbs violently, which action is generally extended to the larger arteries, and indeed may be seen and felt in the aorta, from the epigastrium to its division, on account of the hard and retracted condition of the abdominal walls; there is an anxious contracted countenance, excessive prostration, loathing of food, a burning sensation in the stomach, a feeling of weight and tenderness at the epigastrium, and most obstinate constipation, or where discharges occur spontaneously, they are dry, scanty, generally in hardened fetid balls, and difficult of extrusion; the secretion of the kidneys is scanty, as is also that of the other secretory organs; the patient feels dejected and despondent. And now for the great diagnostic sign of the disease: it is a peculiar odor which arises from the breath, skin, urine, and all the excretions of the body. Upon first perceiving it in the breath, one might suppose his patient was badly salivated, as it partakes much of the character of the odor from ptyalism; still there is a peculiarity about it which one acquainted with the disease will never mistake. Dr. BYFORD has most aptly and successfully hit upon the following comparison: "It resembles almost exactly a strong odor of chloroform, mingled with the smell of animal secretions; say a smell of chloroform and the breath of a patient salivated, and we would have it almost precisely." This odor proceeds principally from the mouth and lungs, and is so disgusting as to make it very disagreeable to be in the same room with the patient; and is so strong as sometimes to be perceived at some distance from the patient's room, and even from the house. Many physicians rely upon it as their almost sole diagnostic sign, and experienced practitioners will know the medicines they will use ere they enter the house. The odor may be perceived in all stages of the disease, but becomes especially marked and powerful as the complaint advances in its course. Should no relief be obtained now, the symptoms I have enumerated above gradually becomes worse; there arises a sense of emptiness in the chest, which the sufferer, with all his efforts—and they are unremitting—is unable to relieve. The efforts at emesis increase, and the matter thrown up becomes darker as the complaint advances; the surface is cold; the breathing slow and irregular; the pulse grows smaller, more rapid, and at last fails, often some hours before death; coma gradually supervenes, and death closes the scene. In other cases, after some days from the commencement, the bowels may act spontaneously, or be moved by a cathartic, when the abdomen becomes swollen, hot, tympanitic, and painful; either watery discharges occur, streaked with blood and mucous floculi, or the passages are hard and fetid; the tongue is coated with a dark fur, and is dry, red, and cracked. Sorded teeth, congested fauces, difficult deglutition, small, quick, weak pulse, cool extremities, retained urine, and coma; the patient generally lying upon his back, with the knees drawn up; death "gradually steals upon him," being immediately preceded by those signs which mark a total prostration of the system, as from a narcotic poison. If the case ends favorably,—after having obtained a few discharges from the bowels, the vomiting becomes less frequent, the sense of burning and weight in the stomach are relieved, the nausea subsides, tongue cleans, appetite improves, and all the symptoms gradually pass away, leaving the patient to a slow convalescence, often accompanied by the promonitory symptoms. Occasionally the convalescence is rapid, and good health is regained in a few days, but, unfortunately, this seldom occurs.

The foregoing is a description of the simple form. The other two varieties are both rare and dangerous, and appear as follows: The inflammatory, as might be supposed, is more fatal than the simple, less so than the congestive variety. It is marked by high arterial excitement; severe headache; the surface of the body is hot, the face flushed, and often active delirium; full, hard, quick pulse; stomach and bowels tender on pressure. It often ends in inflammation of stomach and bowels, and may be complicated with the diseases prevailing at the time in the neighborhood, which complaints often act as an exciting cause. The congestive variety is the most rare and most fatal of the three, equalling in mortality the most dangerous of the various congestive fevers of the West. It is supposed, on good authority, that the disorder commonly assumed this form in the country's early history, as Milksickness was then considered one of our most fatal diseases. After the symptoms of the premonitory stage have continued for a longer or shorter period, a sense of impending suffocation, and intense vomiting and retching set in, accompanied by insatiable thirst, anxiety, cold extremities, small, weak, frequent pulse; glassy appearance of the eyes, abundant watery perspiration, jactitation, prostration, restlessness, suppressed urine, and delirium. These signs mark the form of the disease. Sometimes coma comes on without change of pulse, with full capillary vessels, and thus ushers the patient into the presence of death. The peculiar odor, and other characteristic symptoms, show the nature of the complaint. This variety attacks all grades of constitutions, but, excepting where the cause is intense, prefers the weak and debilitated. The diagnosis of this complaint is, to the experienced physician, comparatively easy and simple. If the peculiar odor proceeding from the patient, the obstinate constipation, dry scybalous fœces, the emesis and matter ejected, the peculiar respiration, craving for ardent spirits, and previous history of the case be properly considered, there need be no mistake in drawing a correct conclusion. And as the reputation of many a good physician has been sacrificed at the side of a Milksick patient, it would be well for one practicing in such a district to make himself as thoroughly acquainted with the disease as possible before he meets it. Our success in practice depends upon the good opinion of the *mass* of the people where we may locate, and the opinion of such a mass is changed by the slightest circumstance which points to our ignorance, though it may arise merely from hesitation or embarrassment, neither of which must be observed by our patrons.

Many diseases resemble this in *some* respects, but the peculiar characteristics enumerated above will enable us to distinguish it from all others. The prognosis varies with the variety of the disease In the simple form it may always be favorable; the patient often recovering without much treatment, always where proper measures are resorted to. Upon the occurrence of inflammatory symptoms, more care must be exercised in expressing an opinion; we may indeed generally pronounce the case doubtful. Where the congestive form arises our prognosis should always be favorable; for though by active and proper measures we may save the patient, the chances are as more than ten to one against us. When death occurs, in the congestive variety, it takes place in from a few hours to three days from the commencement of the attack; in the other forms, from two or three days to two weeks intervene between the attack and the close of the conflict.

The treatment is plain, simple, but active. After the stomach is quieted, the bowels moved, and secretions restored, little is to be done, aside from attention to the general health. For restoring the stomach to quietude, we must rely mainly upon blisters and sinapisms to the epigastrium, and friction with oil of turpentine on the spinal column, combined with small pellets of ice taken internally. Many practitioners have immediate recourse to opium and its preparations, but in consequence of its strong constipation tendencies, and inclination to check the secretions, I would not recommend its use, as we have other remedies quite as potent and less injurious. Some of the aromatic mixtures may be useful, especially if combined in some proportion with brandy; or brandy or whiskey alone may be used; although where other remedies will succeed it is better not to meddle with spiritous liquors, especially in the large quantities recommended by some, as many of the genus homo are possessed of comparatively short reasoning powers, and consequently conclude that whiskey saved their lives—therefore it is a "bully thing," and having once taken hold of their affections, draws them from every other object, and finally lands the subject in the grave. The effervescing draft is a very efficacious remedy under the circumstances, and combined with sweet spirits of nitre, is perhaps excelled by no other, and in its use three indications are fulfilled at once: the stomach is calmed, diaphoresis is produced, and the kidneys aroused to action; also some effect is often induced upon the intestines. Where the vomiting still continues obstinate, and nausea is excessive, recourse may be had to creosote with every hope of success. The late Dr. TRAFTON used yeast, in this stage, with great success, and attributed to it peculiar curative powers in this complaint. Where one of these remedies fails we must turn to another, until we find one to answer our purpose, which we almost certainly will amongst the many in use. But we must not wait for the gastric irritation to subside before we attempt to move the bowels, or we will certainly see our patient soon out of reach. As soon as the first attack of vomiting, after we see the case, subsides, the patient should take a small dose of calomel and jalap, say two grains of each, repeated every hour, or calomel 2 gr., ipecac ¼ gr., same way until eight or ten doses have been taken; when it may be changed for a seidlitz powder combined with one drachm sulph. magnesia, given as frequently, until an operation is produced, or the delay becomes dangerous. The calomel is useful, not only in arousing the torpid liver, but in quieting the stomach, and producing a general alterative effect upon the various secretory functions; but great care should be taken that it is not carried to excess and ptyalism produced. The jalap will aid it much in its cathartic properties, and will restore the dry, irritating fœces to a fluid condition; where ipecac is used instead, in very small doses, it gives tone to the stomach, and assists much in quieting that organ, at the same time having a diaphoretic tendency. When there is an objection to the calomel at first, other cathartics may be used until the tube is opened, when one or two full doses of the submur. or pill. hy-drarg., with salts or oil at a proper interval, should certainly be given, as the liver will properly act under no other remedy, unless I except the podophyllin, which needs further trial. Cream tartar and syrup sennæ, epsom salts, or any other good purgative may be given, a full dose every hour until some effect is produced; it would be well to change from one to another, as being more agreeable to the patient, and often a combination of different remedies, or a change from one to another will produce a more speedy effect. The best time to give medicine is immediately after the ejection of the contents of the stomach; as before it can be aroused to another action, some of the medicine will be absorbed; however little that may be "every little helps," and we will finally see the good results of perseverence. Along with the remedies above proposed, small doses of nit. potassa, or spirits nitre, may be given at intervals of one or two hours. I give this for its tendency to the skin and kidneys. But our great effort must be to obtain a downward passage through the alimentary canal, and that should be accomplished within twenty-four hours; before that time we need labor under no anxiety, unless the symptoms become much worse. As soon as we perceive a peristaltic action induced, which may be known by the improved appearance of the abdominal walls, borborygma, and feelings of the patient, and will occur in from twelve to eighteen hours, it will expedite matters much to administer an injection composed of a solution of sulph. magnesia in warm water, or castor oil floating in water; and if a strong impression is desired, a little ol. terebinthinæ may be added, but as a general thing it is too drastic, and the others answer the purpose well. The first evacuations will be dark and fetid and tarry in consistence, where injections have not been used; where they have they will be thinner, but most disgusting to sight and smell.

Where difficulty is experienced in moving the bowels, we must resort to stronger measures; and here the two great remedies are croton oil and elaterium; the former may be administered according to Dr. BACON'S plan, which is at once ingenious and effectual: a drop of oil is deposited in the centre of a large bolus of the pil. hydrarg., so large as by its weight and size to prevent its ejection; this may be repeated in an hour if no effect has been produced. The elaterium is a powerful remedy, and in small repeated doses will not disappoint our trust, although from the slight intestinal irritation it is rather contraindicated. Podophyllin is also an excellent purgative, possessing also some alterative properties, and should receive a trial. After the constipation has been overcome by a free fœcal discharge, the patient quickly improves in feelings and appearance; the nausea subsides, the skin becomes clearer, the tongue cleans, and the general functions of the system enter again upon a discharge of duty.

Now our attention must be mainly directed to the general health. In order to obviate the tendency to constipation which remains, we must resort daily to laxatives and apperients; amongst the many are rhubarb, magnesia, aloes, etc.; a good combination is one mentioned by Dr. Byford, viz.: rhubarb, aloes, and capsicum, equal parts, and made into a pill. Another excellent preparation is a powder composed of one part bi-tartrate potassa, and two parts of sulphur; sulphur indeed seems to possess a very potent influence over the disease itself, acting as a decided eliminative. Should the stomach lack tone, and the appetite be poor, we may employ quinine, the chalybeates, or bitter infusions; quinia in full doses, after the disease is crushed, may be employed with advantage, and when the patient comes fully under the influence keep him there some days. By this treatment, with plain nutritious diet, the patient, in a shorter or longer time, varying from a few days to three or four weeks, becomes quite convalescent and enjoys good health; where the disease returns or continues obstinate, some vital point in the treatment has been omitted. Sometimes after the first fœcal evacuations are obtained, the stomach continues irritable, the epigastrium and abdominal regions are tender, the latter tumefied, and the discharges from the bowels become frequent and watery; here we have to fear intestinal inflammation, and should endeavor to counteract it by a sinapism to the stomach, cooling mucilaginous drinks, pellets of ice, and injections which while being laxative should also be soothing and emollient. Where great debility and prostration ensue, resort must be had to stimulants, as carb. ammonia, with hot flannels, and frictions or rubefacients to the extremities; spirituous liquors may here become necessary, but as I remarked above, use them with caution, and as little as possible. Blood may be drawn if the case is inflammatory, the indications call for it, and the patient is plethoric; but as a general rule the system will not endure it, and as much strength as possible should be saved for the convalescence; veratrum viride answers the full purpose of venesection, with none of its objections, and should the stomach not be able to retain it, I should not hesitate, the febrile action being high, to administer it per anum.

In the congestive variety, our treatment must be prompt, thorough, and active; large injections of warm water, or in very prostrate cases, water containing ol. terebinth., or brandy should be immediately thrown into the colon; at the same time sinapisms, or friction with hot oil of turpentine, should be applied to the spine and extremities; bottles of hot water, hot cloths, bricks, or irons may be used; cloths wrung out of hot water are very useful; a good and quickly prepared rubefacient is a sack or bag containing common salt and made quite hot; this may be applied to the limbs and body of the patient with great advantage; carb. ammonia, warm brandy or whiskey will be useful, per orem; where the head is warm ice should be applied to it; if the body is in the same condition, apply to it cloths wrung out of cold water, keeping the parts constantly cool, and omitting the remedies per orem as their effect may be injurious. Palliatives, such as lime water, soda water, lemonade, chalk, etc. may be used during the course of the disease, if not contraindicated, as they are generally very grateful to the patient; large draughts of cold water should be forbidden throughout the treatment, especially at the beginning, as they never fail to increase the vomiting; if there is excessive thirst, give rather the warm herb infusions, as the catnip, pennyroyal, or mint teas; or what is better still, infusion of cimicifuga rac. or wild cherry bark.

I have endeavored, gentlemen, to give as full and complete an account of this disease as "in me lies." If I have but partly succeeded in stating the *whole* case, still I must be content, hoping ere another student writes Milksick for his thesis, the profession will give him more solid ground upon which to work, especially in its ætiology.

---

LITHOTRITY PERFORMED ON KING LEOPOLD.—M. CIVIALE has lately performed lithotrity on King Leopold of Belgium. The calculus was small, and was crushed successfully in two sittings. Some time since M. C. performed the some operation for King Bomba, for which he received a fee of 25,000 dollars.

